# JavaCyte, a novel open-source tool for automated quantification of key hallmarks of cardiac structural remodeling

**DOI:** 10.1038/s41598-020-76932-3

**Published:** 2020-11-18

**Authors:** J. Winters, M. Edler von Braunmuhl, S. Zeemering, M. Gilbers, T. Ten Brink, B. Scaf, E. Guasch, L. Mont, M. Batlle, M. Sinner, S. Hatem, M. K. Mansour, L. Fabritz, L. C. Sommerfeld, P. Kirchhof, A. Isaacs, M. Stoll, U. Schotten, S. Verheule

**Affiliations:** 1 Department of Physiology, Cardiovascular Research Institute Maastricht, Universiteitssingel 50, P.O. Box 616, 6200 MD Maastricht, The Netherlands; 2 Cardiovascular Institute, Hospital Clinic de Barcelona, Barcelona, Spain; 3 Institut D’Investigacions Biomédiques, August Pi I Sunver (IDIBAPS), Barcelona, Spain; 4 CIBERCV, Madrid, Spain; 5 INSERM UMRS1166, ICAN - Institute of CardioMetabolism and Nutrition, Sorbonne Université, Institut de Cardiologie, Hôpital Pitié-Salpêtrière, Paris, France; 6 Hôpital Necker Enfants Malades, Paris, France; 7 Institute of Cardiovascular Sciences, University of Birmingham, Birmingham, UK; 8 Departments of Cardiology, University Hospital Birmingham, Birmingham, UK; 9 Department of Cardiology, SWBH NHS Trust, Birmingham, UK; 10 University Heart and Vascular Center, UKE Hamburg, Hamburg, Germany; 11 German Centre for Cardiovascular Research (DZHK), Hamburg, Germany; 12 Department of Biochemistry, Cardiovascular Research Institute Maastricht, Maastricht, The Netherlands

**Keywords:** Cardiovascular diseases, Heart failure, Atrial fibrillation

## Abstract

Many cardiac pathologies involve changes in tissue structure. Conventional analysis of structural features is extremely time-consuming and subject to observer bias. The possibility to determine spatial interrelations between these features is often not fully exploited. We developed a staining protocol and an ImageJ-based tool (JavaCyte) for automated histological analysis of cardiac structure, including quantification of cardiomyocyte size, overall and endomysial fibrosis, spatial patterns of endomysial fibrosis, fibroblast density, capillary density and capillary size. This automated analysis was compared to manual quantification in several well-characterized goat models of atrial fibrillation (AF). In addition, we tested inter-observer variability in atrial biopsies from the CATCH-ME consortium atrial tissue bank, with patients stratified by their cardiovascular risk profile for structural remodeling. We were able to reproduce previous manually derived histological findings in goat models for AF and AV block (AVB) using JavaCyte. Furthermore, strong correlation was found between manual and automated observations for myocyte count (r = 0.94, *p* < 0.001), myocyte diameter (r = 0.97, *p* < 0.001), endomysial fibrosis (r = 0.98, *p* < 0.001) and capillary count (r = 0.95, *p* < 0.001) in human biopsies. No significant variation between observers was observed (ICC = 0.89, *p* < 0.001). We developed and validated an open-source tool for high-throughput, automated histological analysis of cardiac tissue properties. JavaCyte was as accurate as manual measurements, with less inter-observer variability and faster throughput.

## Introduction

The progression of multiple cardiac diseases causes, or is associated with, gradual changes of cardiac tissue structure. These structural alterations alter the passive mechanical properties of the myocardium, contractility, electrical propagation, tissue perfusion and energetics. Quantification and comparison of cardiac tissue structure normally relies on manual inspection and measurements by researchers, making the process time-consuming and subject to inter-observer variability. Therefore, we developed a suite of algorithms for automated quantification of structural features in myocardial tissue.

**Figure 1:**
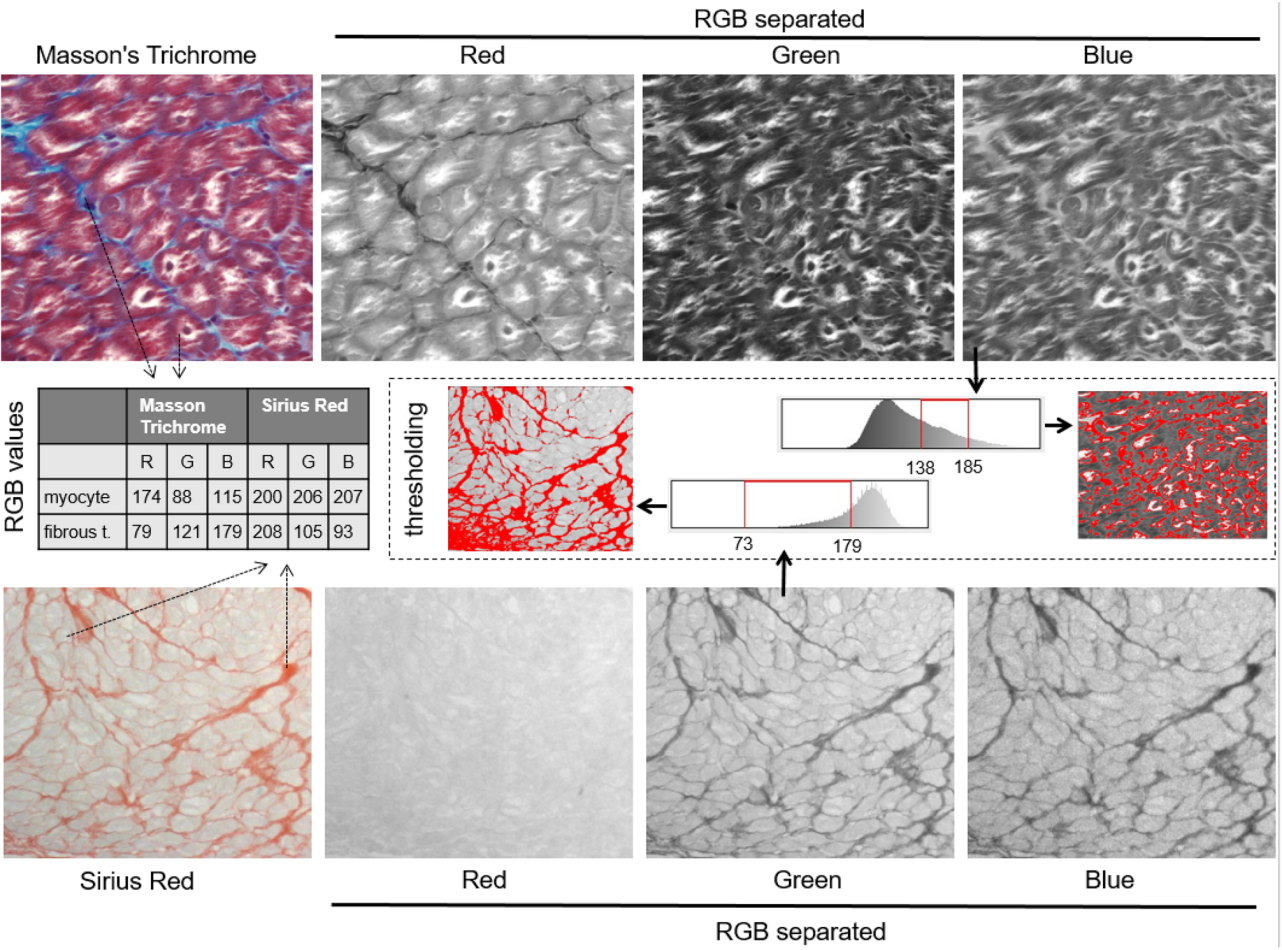
Disadvantages of automated analysis of Sirius red and Masson’s Trichrome staining. Similarity in RGB values for myocytes and fibrous tissue following Sirius red staining complicate automated analysis. Manual thresholding is slow, inaccurate and prone to observer bias. Endomysial septa are poorly visibile in both staining approaches. Co-localization of other important markers of structural remodeling is not possible.

A key factor in myocardial remodeling is fibrosis, an increase in the amount of fibrous tissue with age that is accelerated by the presence of chronic cardiovascular diseases. Increased fibrosis has been reported in left ventricular failure^1^, right ventricular dysfunction^2^, and atrial fibrillation (AF)^3^, among other conditions. Seminal work by Weber and co-workers described several distinct types of fibrosis, which are caused by different disease processes^4^. Reparative fibrosis, also called replacement fibrosis, occurs in reaction to myocyte death, e.g. after myocardial infarction, typically leading to larger areas of fibrous tissue deposition. By contrast, reactive fibrosis, also called interstitial fibrosis, occurs in reaction to increased stretch, pressure or volume overload or neurohumoral activation. Interstitial fibrosis can lead to thickening of collagen septa around bundles of myocytes (perimysial fibrosis) and between myocytes within bundles (endomysial fibrosis). Endomysial fibrosis leads to increased cell-to-cell distances between myocytes and can thereby impair electrical propagation^5,6^. In a goat model, we previously demonstrated that 6 months of AF does not lead to an increase of overall fibrosis but does result in endomysial fibrosis in the epicardial layer of the atria, impairing transverse propagation^6^. In the ventricles, increased endomysial fibrosis is viewed as a key determinant of increased ventricular stiffness leading to diastolic heart failure^7,8^. Assessment of fibrosis is commonly performed in histological sections by quantifying the relative area occupied by fibrous tissue in Sirius Red or Masson's Trichrome staining, irrespective of the particular type of fibrosis. This usually involves setting a subjective color threshold by the operator to distinguish fibrous tissue from myocytes. However, as is apparent in Fig. 1, there is considerable overlap in the RGB values of myocytes and fibrous tissue, both in Sirius Red and Trichrome staining. As a result, the quantification is sensitive to inhomogeneous illumination (i.e. shading artefacts) and observer bias. Reported values for the percentage of fibrosis in normal, healthy atria vary widely, also within the same species Moreover, whereas larger areas of fibrosis are clearly stained, endomysial septa are not. An alternative for specifically quantifying endomysial fibrosis, the manual measurement of cell-to-cell distances, is extremely time-consuming^6^. In the context of fibrosis, the density of fibroblasts is also relevant^9^, both because they are responsible for excess extracellular matrix ECM formation, and because fibroblasts may couple to myocytes electrically, thereby affecting conduction through electrotonic interactions^10,11^.

Another hallmark of structural remodeling is myocyte hypertrophy. Increased myocyte size relates to increased wall thickness and chamber dilatation macroscopically, and can also alter electrical propagation, both in the ventricles^12^ and in the atria^13,14^. Again, manually measuring myocyte dimensions in histological or immunohistochemical staining is time-intensive and prone to observer bias.

Capillary rarefaction has also been linked to myocardial dysfunction, such as in heart failure with preserved ejection fraction (HFpEF) and AF^15,16^. Capillary density is often reported as the number of capillaries per unit of surface area. Although this is a relevant correlate of oxygen diffusion distances, it does not accurately reflect the loss or gain in number of capillaries in settings with concomitant myocyte hypertrophy. In those circumstances, the number of capillaries per myocyte is needed to assess the degree of capillary rarefaction or angiogenesis.

We developed a fluorescent triple staining method and an integrated suite of algorithms for the automated quantification of all of the aforementioned structural features. Wheat Germ Agglutinin (WGA) was used to visualize the extracellular matrix (ECM)^17^, yielding the number and size of myocytes, overall fibrosis and myocyte-to-myocyte distances as a measure for endomysial fibrosis. Capillaries were stained with the biotinylated isolectin Griffonia Simplifolia (GS-IB4) in animal tissues and CD31 in human biopsies, providing the size and number of capillaries. Fibroblasts were stained with anti-vimentin antibody, yielding the size and distribution of fibroblasts. From these data, the number of capillaries or fibroblasts can be calculated in a simple manner. Because the positions of all recognized structures are stored, this method also allows analysis of the spatial correlation between various parameters. This method was successfully tested in atrial tissue samples from goats, pigs, horses and humans. Here, we present the validation of the method in several goat models of AF that were previously characterized extensively by manual analysis. In addition, we analyzed biopsies from selected patient populations. In general, the extent of atrial pathology is far larger in patient samples than in animal models of heart disease. For this reason, we analyzed the degree of inter-observer variability in these human biopsies as a test case. For these data, we also extended our analysis to spatial heterogeneity in the distribution of endomysial fibrosis, thereby providing an example of the potential for further development.

## Materials and methods

### Tissue sampling

Collections of atrial tissue biopsies from several previously described and characterized goat models were used: sham-operated goats (n = 6), goats with AF maintained by burst pacing for 3 weeks (short term, ST, n = 7) and 6 months (long term, LT, n = 6)^18^ and goats with atrial dilatation as a result of His bundle ablation (AV block, AVB, n = 6)^19^. All animal experiments were approved by the ethical board for animal experimentation ‘Centrale Commissie Dierproeven (CCD)’ and were in compliance with the European directive 2010/63/EU on the protection of animals used for scientific purposes. All research on human atrial tissue samples was performed in accordance with relevant guidelines, as approved by the ethical committee "METC academic hospital Maastricht/university Maastricht". Informed consent was obtained from all participants.

Human atrial samples (n = 14 left atrial appendages, LAA, and n = 8 right atrial appendages, RAA) were collected from the CATCH-ME atrial tissue biobank from patients undergoing coronary artery bypass grafting (CABG), valvular surgery or heart transplant surgery (Table 1). Patients were stratified based on the expected risk for the presence of atrial structural remodeling associated with their clinical profile. Cardiovascular risk profile positive patients (CVRP+, n = 11) were all diagnosed with both heart failure and persistent AF. Tissue from all but one of the patients in this group was obtained during a heart transplant xenograft surgery. The remaining patient was diagnosed with grade III mitral valve regurgitation. Most (n = 6) of the CVRP+ patients had an underlying valvular cardiomyopathy, predominantly resulting from the mitral valve. In two other CVRP+ patients, the cardiomyopathy was ischemic. None of the patients had a known genetic basis for cardiomyopathy, long QT syndrome or Brugada syndrome. Two CVRP+ patients, however, were diagnosed with an idiopathic non-obstructive cardiomyopathy. As a whole, ejection fractions were significantly lower in the CVRP+ group, resulting in a higher NYHA classification. Cardiovascular risk profile negative patients (CVRP–, n = 11) had no history of either AF or heart failure. In seven CVRP– patients, the primary indication for surgery was coronary artery bypass grafting (CABG). Three others had grade I or II mitral valve regurgitation, requiring mitral valve repair. One tissue sample was collected from a vegetative donor. Patients from the CVRP+ group were matched to CVRP– patients based on sampling side (LAA/RAA), gender (exactly matched) and age (± 5y).

**Table 1:** Distribution of clinical parameters of patients with and without a cardiovascular risk profile for structural remodeling (CVRP).

	CVRP −	CVRP+	*p* value
Age (yrs)	64 (± 7.6)	63 (± 5.8)	0.66
Sex (F/M)	1/10	1/10	1.00
BMI	26 (± 3.7)	27 (± 3.8)	0.54
Rhythm (pers. AF)	0 (0%)	11 (100%)	< 0.001
Heart Failure	0 (0%)	11 (100%)	< 0.001
EF (%)	61 (± 10)	30 (± 12)	< 0.001
NYHA	1.3 (± 1.2)	2.8 (± 1.3)	0.01
Indication for surgery			< 0.001
CABG	7 (64%)	0 (0%)	
Donor	1 (9%)	0 (0%)	
HTX	0 (0%)	10 (46%)	
MVD	3 (27%)	1 (9. %)	
Hypertension	6 (55%)	6 (55%)	1.00
Location (LAA/RAA)	7 (64%)/4 (36%)	7 (64%)/4 (36%)	1.00

A chi-square test of independence is used for categorical variables, and a t-test for continuous variables.

*BMI* body mass index, *EF* ejection fraction, *CABG* coronary artery bypass graft, *HTX* heart transplant xenograft, *MVD* mitral valve disease, *LAA/RAA* lef/right atrial appendage.

### Triple staining

**Figure 2:**
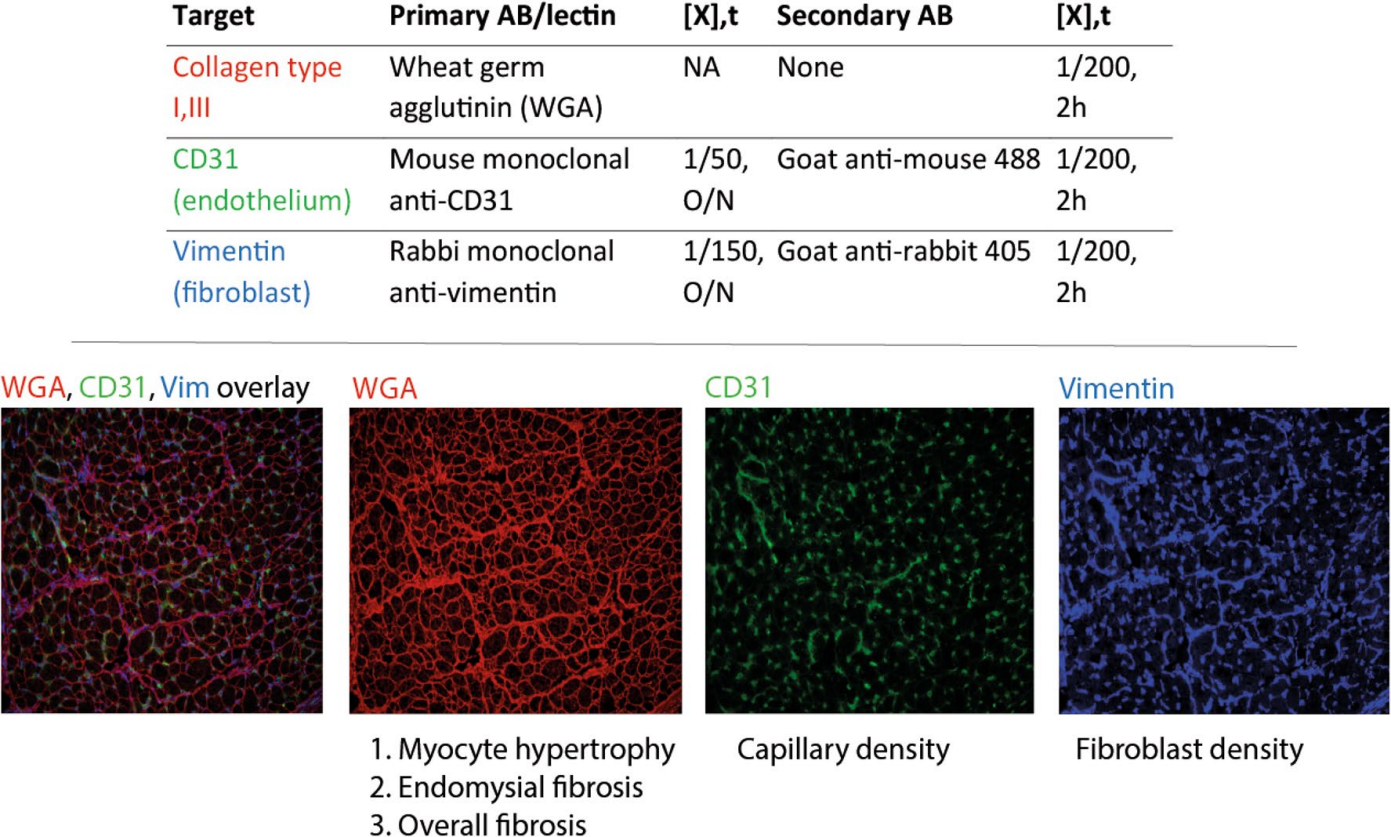
Triple immunohistochemical staining for the visualization of fibrous structures, endothelial cells and fibroblasts. Optimal contrast between three fluorescent components allows to study the spatial correlation between fibrous tissue (red, WGA), endothelial cells (green, CD31/GSI-B4) and fibroblasts (vimentin).

A complete and detailed protocol for the staining method is provided in supplement 1. All atrial tissues were excised rapidly, snap frozen and stored at − 80 °C. Cryosections of 6 µm were made at − 22 °C and stored at − 80 °C. Antigen fixation was performed by submerging slides in ice-cold acetone for 10 min. Nonspecific binding sites were blocked for 60 min with a blocking solution consisting of 2 m/v % fraction V BSA and 0.3 M glycine dissolved in PBS. Fibroblasts were visualized with an overnight stain using a mouse monoclonal anti-vimentin antibody at a concentration of 1/150. In human samples endothelial cells were stained overnight at room temperature with a monoclonal anti-human CD31 antibody at a concentration of 1/50. The next morning, sections were incubated for 120 min at room temperature with appropriate fluorescent secondary antibodies at a concentration of 1/200 (Fig. 2). Cell membranes were stained at room temperature using WGA lectin conjugated to an Alexa 594 fluorophore at a 1/200 dilution. In animal tissue, capillaries were labeled with the biotinylated GS-IB4 conjugated to Alexa Fluor 488 at a dilution of 1/200, for a duration of 120 min. Coverslips were mounted with 'Prolong Gold Antifade' mounting medium (ThermoFisher Scientific). Stained sections were stored at 4 °C prior to microscopy.

### Microscopy

Sections were imaged using a Leica DM4B microscope with a Leica MC170 HD camera. WGA, GS-IB4/ CD31 and vimentin were visualized using Leica filter cubes A (bandpass, BP, 340–380 nm), N2.1 (BP515–560 nm) and I3 (BP450–490 nm), respectively. Only areas where myocytes were sectioned approximately transversely were selected and photographed for further analysis.

### Automated quantification

To perform a standardized, automated analysis of structural remodeling, JavaCyte was developed for the public domain image analysis platform Fiji (www.fiji.sc), an extended version of ImageJ. Output files with the position and dimensions of all structures recognized by the algorithms are stored as comma separated value (csv) files, compatible with most statistical packages, for further analysis. The code of JavaCyte was made available as an open source at GitHub in the repository stored under: https://github.com/jwintersUM/JavaCyte.

### Fibrosis, myocyte size and myocyte dissociation index, clustering coefficient

**Figure 3:**
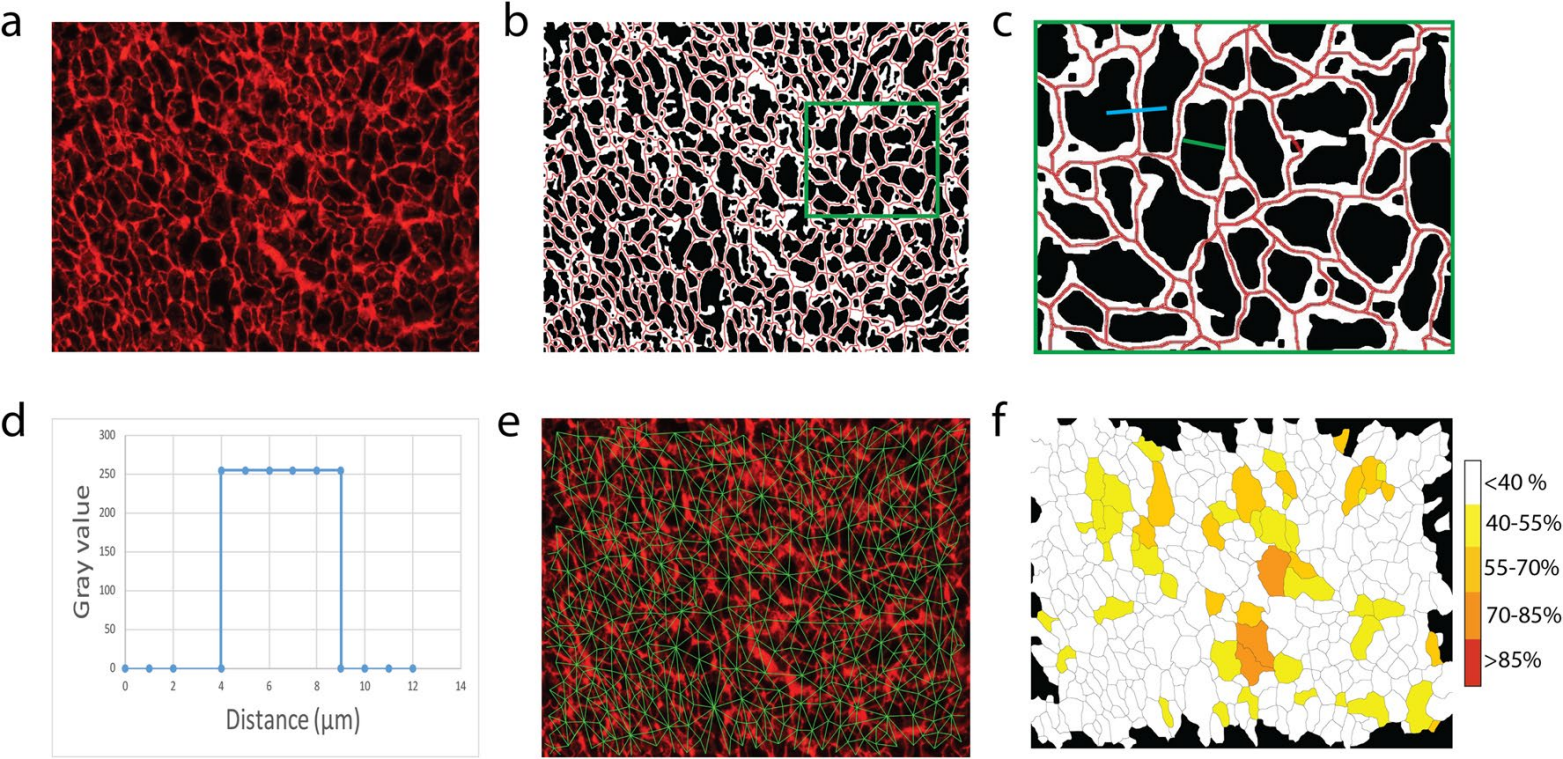
Analysis pipeline of WGA images applying JavaCyte. (**a**) Original image following WGA staining. (**b**) Phansalkar thresholding of a WGA image. ECM is represented in white, cardiomyocytes in black. (**c**) For each cardiomyocyte, minimal Feret diameter (green) is determined as a measure for size. Neighbors are detected. Local minima of neighboring cells are connected with a line selection (yellow). (**d**) Pixel values across the line selection are plotted. The width of the profile plot corresponds to the width of the endomysial septum between two neighboring cardiomyocytes. (**e**) A control image is generated for visual inspection of accuracy. (**f**) For each cell, cardiomyocyte dissociation index (CDI) is visualized as a measure for fibrosis surrounding the cell.

A Phansalkar’s algorithm for automatic local thresholding was used to delineate WGA-positive pixels (Fig. 3a,b). The total area of fibrosis was calculated as the fraction of WGA-positive pixels to the total pixel count. Next, the original WGA-images were filtered using a Gaussian blur function to reduce noise. For each image, the average contrast between WGA-positive and -negative pixels was used to obtain the 'optimal prominence' setting for cardiomyocyte detection using Fiji’s built-in 'segment particles' function to determine local minima in fluorescence. Most of the recognized local minima are myocytes, but some local minima represent small dark patches within larger regions of fibrosis. To reduce the number of false positive structures, the pixel intensity directly surrounding local minima was determined. Local minima for which the surrounding pixel values where in the red spectrum were rejected as a cardiomyocyte and were masked. For the remaining local minima, Voronoi watershedding and particle analysis was applied. Myocyte size for each object was calculated as the minimal Feret diameter. For each detected myocyte, all directly neighboring myocytes were defined. A profile plot of the line selection between the local minima of each pair of neighboring myocytes was generated. The thickness of the ECM in between myocytes (i.e. endomysial collagen septa) was calculated as a measure of endomysial fibrosis (Fig. 3c–e). Manual inspection of output images generated by JavaCyte (Fig. 3e) is required to guarantee optimal accuracy. In these output images, a thresholded version of the measurements taken is shown as an overlay with the original image, so the user can ascertain the accuracy of the analysis.

As an extension of the method in human samples, a cardiomyocyte dissociation index (CDI) was obtained for each cell by determining the fraction of inter-cardiomyocyte distances that exceeded the mean value measured in CVRP– patients with an indication for CABG surgery, i.e. the 'healthiest' patients in our study population, by more than two standard deviations. Distribution of CDI was visualized for each image as a heat map (white cells are closely apposed to neighbors while dark red cells are isolated from neighbors, Fig. 3f). Furthermore, clustering of enhanced inter-myocyte distances was tested in analogy with the local clustering coefficient, as described in graph theory. Myocytes separated by a larger than normal area of endomysial fibrosis are connected in a clique. The clustering coefficient of myocytes in a clique is enhanced. The impact of augmented clustering coefficients in a clique directly corresponds to the size of the clique. The average clustering coefficient of all myocytes in an image was measured using Matlab. Permutation testing was applied to calculate the expected clustering coefficient in the case of a completely random distribution of inter-myocyte distances measured in the image. The mean of 1000 permutations defined the expected clustering coefficient. Furthermore, the proportion of permuted clustering coefficients smaller than the observed clustering coefficient was defined.

### Capillary density and size

**Figure 4:**
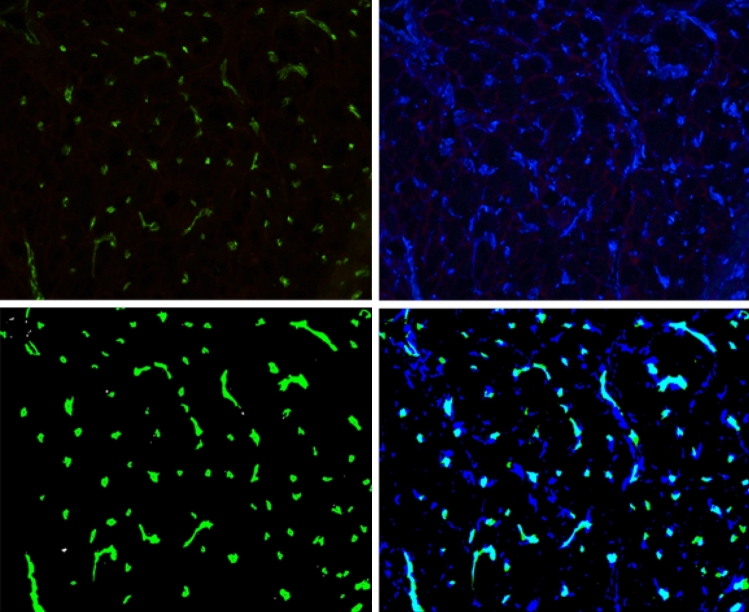
Analysis pipeline of CD31/GSI-B4 and vimentin images applying JavaCyte. CD31/GSI-B4 positive objects (top left) and vimentin positive objects (top right) are segmented (bottom left and bottom right) according to Landini’s algorithm for HSB thresholding. Capillary count is determined. Capillary size is obtained for objects that meet circularity requirements. Non-circular objects are ignored in size measurements, but are counted for determination of the total number. Fibroblast count is obtained after correction for cross-reactive endothelial vimentin staining.

The number of capillaries was obtained by counting CD31 or GSIB4-positive structures using a particle analysis function after applying a Gaussian blur and Landini’s^20^ Hue-Saturation-Brightness (HSB)-based color threshold. A minimal size threshold of 1.5µm^2^ was used to reduce noise. For each image, capillary density was calculated as the number of capillaries per myocyte. For the determination of capillary size, a circularity filter of 0.35 was used to select transversely cut capillaries prior to calculations of the minimal Feret diameter (Fig. 4).

### Fibroblast density

Vimentin positive objects were selected applying Landini’s color threshold^20^. Vimentin shows some cross-reactivity to endothelial cells^21^, which was confirmed by overlaying thresholded GS-I4B/ CD31 and vimentin images. For analysis of fibroblast density, we therefore subtracted the thresholded GS-I4B/ CD31 images from the thresholded vimentin images. For each image, the number of fibroblasts per myocyte was calculated as a measure for fibroblast density (Fig. 4).

### Validation and performance measures

Performance measures were obtained by comparing automated results to data obtained through manual segmentation and measurement. In a first stage, object recognition was tested by quantifying the number of true positive (TP), false positive (FP) and false negative (FN) objects in human atrial samples. True positive denotes the number of manually identified myocytes or capillaries that were recognized by the algorithm. False negative depicts the number of manually identified structures that were not recognized by the algorithm. Performance was analyzed by calculation of sensitivity, precision, false negative rate (FNR) and F1 score, defined by the following formulas:

$$ {\text{Sensitivity}} = { }\frac{{{\text{TP}}}}{{{\text{TP}} + {\text{FN}}}} $$

$$ {\text{Precision}} = { }\frac{TP}{{TP + FP}} $$

$$ FNR = { }\frac{FN}{{FN + TP}} $$

$$ F1 = { }\frac{2TP}{{2TP + FP + FN}} $$

Normal distribution of count data was confirmed with a Shapiro–Wilk’s test. The assumption of homoscedasticity was tested by obtaining a spread-versus-level plot in the statistical program R. Next, correlation between the manually obtained and automated count for myocytes and capillaries was tested applying Pearson’s correlation test. Further validation of optimal object recognition included a second stage where area and minimal Feret diameter of matched truly positive objects was compared. Each manually detected myocyte was assigned to an automatically detected myocyte based on the minimal distance between centroids (analogous to maximal matching described by graph theory), as measured by the following formula:

$$ d = \sqrt {\Delta Cx^{2} + \Delta Cy^{2} } $$

The percentage of deviation of the area and minimal Feret values of automatically recognized objects from manually obtained measurements was calculated and plotted in a Bland–Altman curve. Once optimal myocyte recognition had been achieved, correlation between median inter-myocyte distances obtained manually and by applying the algorithm was tested.

### Inter-observer variation

Sampling variation between two observers was quantified by independently taking 10 photographs per cardiac sample of 10 unique patients. Further analysis was carried out using JavaCyte. Variation in inter-myocyte distance was visualized per sample. To compare observers, a two-way mixed, absolute agreement intra-class correlation test was performed.

### Statistics

The open source software environment for statistical computing, ‘R’ (www.r-project.org), was used for all statistical analysis. For each tissue sample, a minimum of three independent images was obtained, while the operator was blinded to the experimental group or patient group. For analysis of endomysial fibrosis, cardiomyocyte size, CDI and capillary size, the median value per image was calculated. Differences were tested using a mixed-effects model. For analysis of goat samples, the models contained the fixed-effect variables ‘sublayer (endocardium/epicardium)’, ‘group (Sham/ST-AF/LT-AF/AVB)’ and the interaction term ‘sublayer*group’. The factor ‘goat study number’ was included as a random-effect variable. For analysis in human samples the models included the fixed-effect factor ‘group (CVRP+/CVRP–)’ and the random-effect ‘matched pair_ID’ to identify matched patients.

## Results

### Workflow

We developed a suite of algorithms for the automated analysis of the previously described features of myocardial tissue structure, JavaCyte, as a plug-in tool for the commonly used image analysis environment Fiji. We recommend that images for analysis are taken at the same magnification (200–400×) and with similar illumination and exposure, preferably using the same microscope and camera for all images. At magnifications below 200×, the resolution of digital images will become inadequate to accurately determine the dimensions of the recognized structures, particularly the thickness of endomysial septae. At magnifications above 400×, fewer structures will be present within an image, increasing possible selection bias of the photographed locations. The program has been successfully tested for jpg, tiff and bmp image formats, but will work in principle for all standard image formats that can be imported into ImageJ. At the start of image analysis, the user is prompted to enter the resolution (in pixels per millimeter). The user can specify entire folders of images to be analyzed per ‘image color’ (red for WGA, green for CD31/GSIB4, blue for vimentin), and one output folder per analyzed parameter. After specification of the desired analysis, quantification of endomysial fibrosis, CDI, total ECM content, myocyte size and count, capillary size and count, and fibroblast count can be performed simultaneously. The program will produce feedback throughout the analysis phase regarding the percentage of images analyzed. As no manual input is needed during the automated analysis phase, the program can run unattended.

In the output folders, one output image is stored for each original image showing thresholded images of the recognized structures (Fig. 3E). For WGA images, the locations where myocyte size and myocyte-to-myocyte distances were measured are indicated. This allows the user to inspect each analyzed image in order to detect potential recognition problems. In addition, numerical data is written to .csv files, with individual measurements of the location and size of the recognized structures. These files can be directly imported for further statistical analysis in e.g. R or SPSS.

### Inter-myocyte distance and myocyte size in goat models of AF

**Figure 5:**
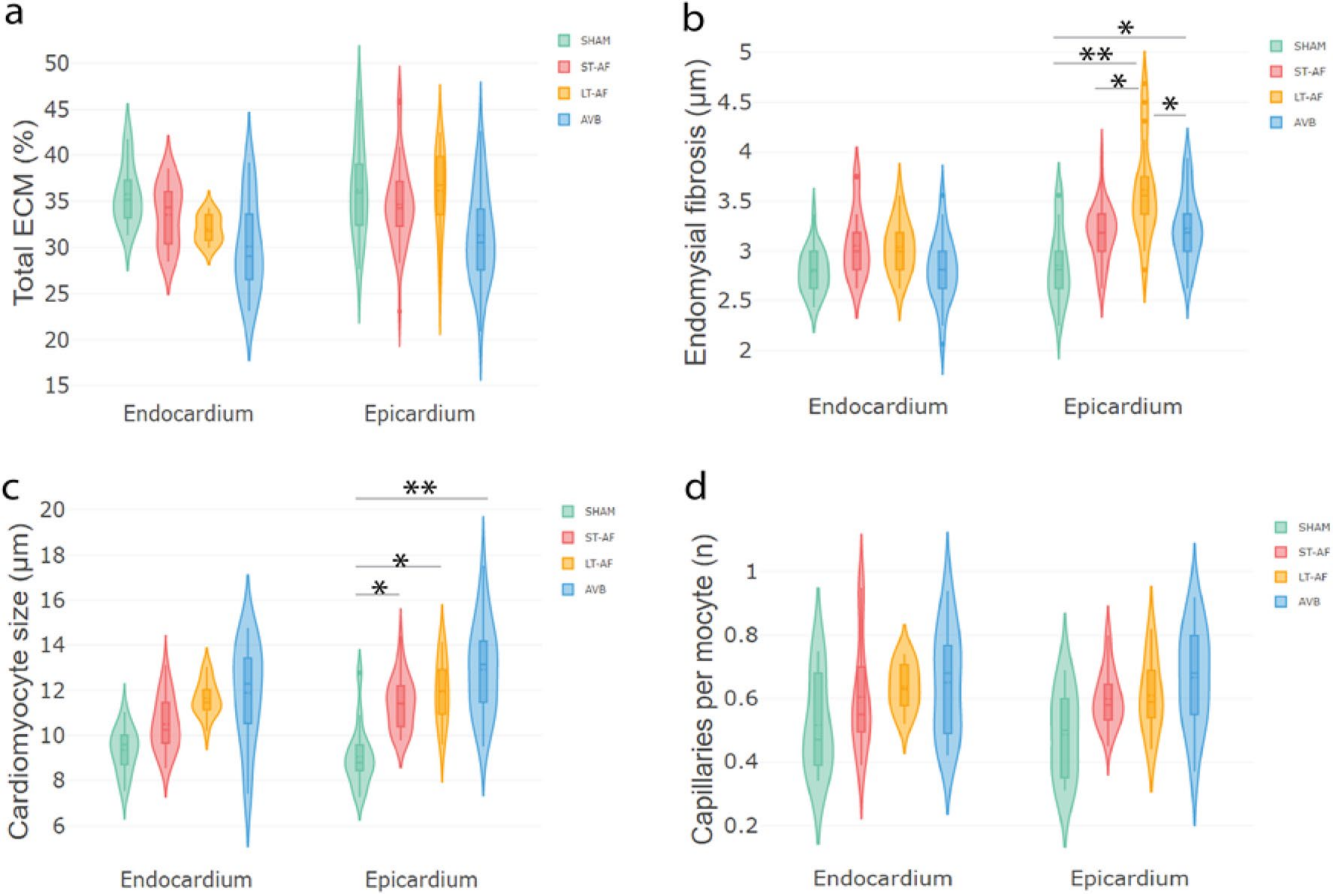
Increased inter-myocyte distance in LT-AF goats and myocyte hypertrophy in LT-AF goats and AVB goats. (**a**) Total ECM content is similar in Sham-operated goats and goats in ST-AF, LT-AF and AVB. (**b**) Significantly more endomysial fibrosis is observed in the epicardial layer in LT-AF goats (3.61 ± 0.10 µm) than in ST-AF goats (3.19 ± 0.08, *p* = 0.01 µm) or Sham-operated goats (2.87 ± 0.10 µm, *p* < 0.001). There is more endomysial fibrosis in AVB goats (3.24 ± 0.09 µm) than Sham-operated goats (2.87 ± 0.10 µm, *p* = 0.02), and more endomysial fibrosis in LT-AF goats (3.61 ± 0.10 µm) than AVB goats (3.24 ± 0.09 µm, *p* = 0.03). These differences are not present in the endocardium. (**c**) Cardiomyocytes are larger in ST-AF goats (11.50 ± 0.48 µm, *p* = 0.03), LT-AF goats (11.72 ± 0.55 µm, *p* = 0.02) and AVB goats (12.83 ± 0.52 µm, *p* < 0.001) compared to Sham-operated goats (9.36 ± 0.57 µm). (**d**) Capillary density or size did not differ. * significant at *p* < 0.05, ** significant at *p* < 0.001.

Structural parameters in the epicardial and endocardial layer from the LA free wall of ST-AF (n = 7), LT-AF (n = 6), AVB (n = 6) and sham-operated (n = 6) goats were studied. In agreement with our earlier quantification in the same model^6^, no significant differences in total ECM content were observed between groups in either of the layers (Fig. 5a). Inter-myocyte distances were analyzed as a measure for endomysial fibrosis. In the epicardial layer, there was significantly more endomysial fibrosis in LT-AF goats than in ST-AF goats (+ 0.42 µm, *p* = 0.01) or Sham-operated goats (+ 0.74 µm, *p* < 0.001). Endomysial fibrosis was increased in AVB goats compared to Sham-operated goats (+ 0.37 µm, *p* = 0.02), and in LT-AF goats compared toAVB goats (+ 0.37 µm, *p* = 0.03).

In addition, bigger myocytes were observed in ST-AF goats (+ 2.14 µm, *p* = 0.03) and in LT-AF goats (+ 2.36 µm, *p* = 0.02) compared to Sham-operated goats. As expected^19^, myocyte diameter was increased in AVB goats compared to Sham-operated goats (+ 3.47 µm, *p* < 0.001).

In the endocardial layer, no changes in inter-myocyte distance (Fig. 5b) or minimal Feret diameters were determined, despite the presence of a trend (Fig. 5c). No changes were observed in capillary density or size in either layer (Fig. 5d), although the number of capillaries per surface area decreased in the endocardium of LT-AF goats compared to sham-operated goats (− 42, *p* = 0.04, results not shown). Fibroblast density was not quantified for these samples. For all parameters analyzed, the direction of change and effect size agreed well between manual and automated quantification (Table 2).

**Table 2:** Manual and automated analysis of cardiomyocyte diameter, inter-myocyte distance and capillary density in goat models for AF and AV block (AVB).

	Cardiomyocyte diameter		Inter-myocyte distance		Capillary density	
	Manual (µm)	JavaCyte (µm)	Manual (µm)	JavaCyte (µm)	Manual	JavaCyte
SHAM	9.69 ± 1.70	9.36 ± 0.57	2.54 ± 0.47	2.87 ± 0.10	0.48 ± 0.13	0.47 ± 0.06
ST-AF	11.83 ± 1.53	11.50 ± 0.48	2.72 ± 0.62	3.19 ± 0.08	0.57 ± 0.02	0.59 ± 0.04
LT-AF	12.65 ± 2.98	11.72 ± 0.55	3.64 ± 0.45	3.61 ± 0.11	0.67 ± 0.02	0.60 ± 0.05
AVB	13.98 ± 1.97	12.83 ± 0.52	3.12 ± 0.39	3.24 ± 0.09	0.68 ± 0.04	0.65 ± 0.04

Values are represented as mean ± SD in µm.

*ST-AF* short term AF.

*LT-AF* long term AF.

### Validation of myocyte recognition, myocyte diameter, inter-myocyte distance and capillary count

**Figure 6:**
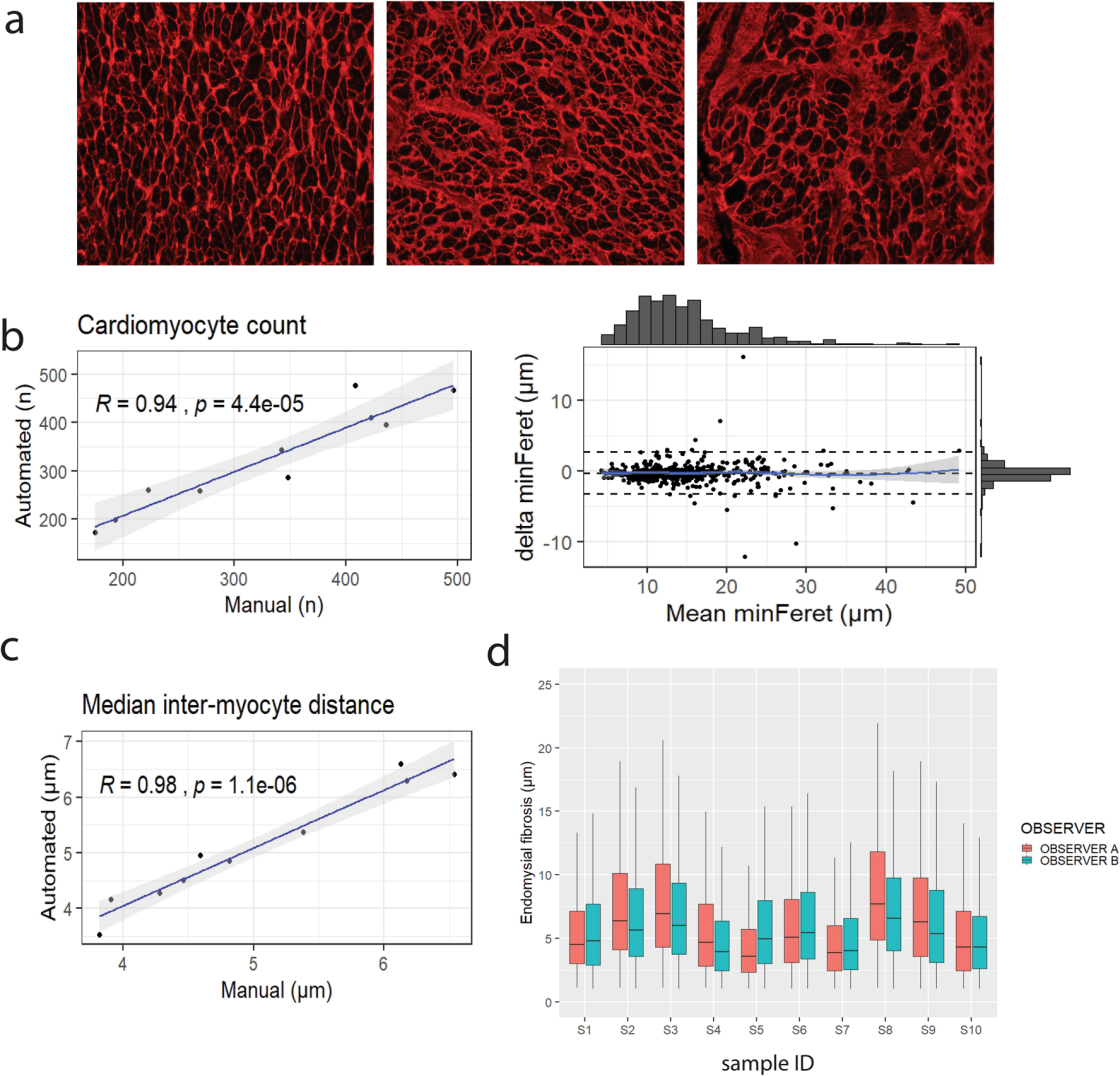
Two-step validation of JavaCyte algorithm. (**a**) Human tissue is typically more heterogeneous resulting from (unknown) comorbidities. (**b**) Manual observations for myocyte count (R = 0.94), correlated strongly to automated results applying JavaCyte. The mean difference in minimal Feret diameter of matched cardiomyocytes is small. (**c**) Manual Median inter-cardiomyocyte distances correlates strongly with measurements made applying JavaCyte. (**d**) Intra-class correlation testing showed no difference between two independent, blinded observers who each acquired 10 unique images per sample of 10 patients (ICC = 0.89).

To validate automated quantification, we compared it to manual quantification of the same images. Pearson’s correlation coefficients were calculated to assess the correlation between automated cardiomyocyte count and manually obtained cardiomyocyte counts from human (n = 10) atrial samples (Fig. 6a,b). A strong positive correlation between both counts was observed (r = 0.94, n = 10, *p* < 0.001). To further evaluate the accuracy of myocyte recognition in human atrial samples, measures of sensitivity (93.9% ± 5.5%), precision (95.4% ± 4.5%), FNR (6.1% ± 5.5%) and F1-score (94.5% ± 3.5%) were calculated. These results show high precision in myocyte detection, while maintaining sensitivity. Next, automatically detected cells were matched with manually recognized cells. Minimal Feret diameter of matched truly positive objects was compared (Fig. 6b). The mean difference between matched manually and automatically obtained cardiomyocyte diameters situates in the 95% confidence interval [− 2.82%, − 1.08%], as shown in a Bland–Altman plot (Fig. 6b). Furthermore, a positive correlation was observed between median (Fig. 6c) inter-myocyte distances measured by the algorithm and manually in human samples (R = 0.98, n = 10, *p* < 0.001). Comparison of the number of capillaries was studied in CD31 images. A Pearson’s correlation coefficient was computed to test the correlation between automated and manually obtained capillary counts, where we also observed a high correlation (R = 0.95; n = 10; *p* < 0.001). Performance measurements in human atrial samples demonstrated high precision (95.3 ± 3.7%) while maintaining considerable sensitivity (92.0 ± 5.2%). Moreover, JavaCyte showed great time-efficiency as manual measurements for total ECM content, inter-myocyte distance, CDI, cardiomyocyte size and count take up to 2 h per image for a trained analyst, while JavaCyte functions unsupervised and analysed 141 images in the same time frame on a standard device with an Intel i7 processor and 32 GB RAM.

The inter-observer effect related to area selection for imaging was quantified. An intra-class correlation test demonstrated reproducibility between both observers (Fig. 6d). A high degree of reliability was found between both observers (ICC = 0.89, n = 10, *p* = 0.001).

### Cardiovascular risk profile as a determinant of atrial tissue structure in patients

**Figure 7:**
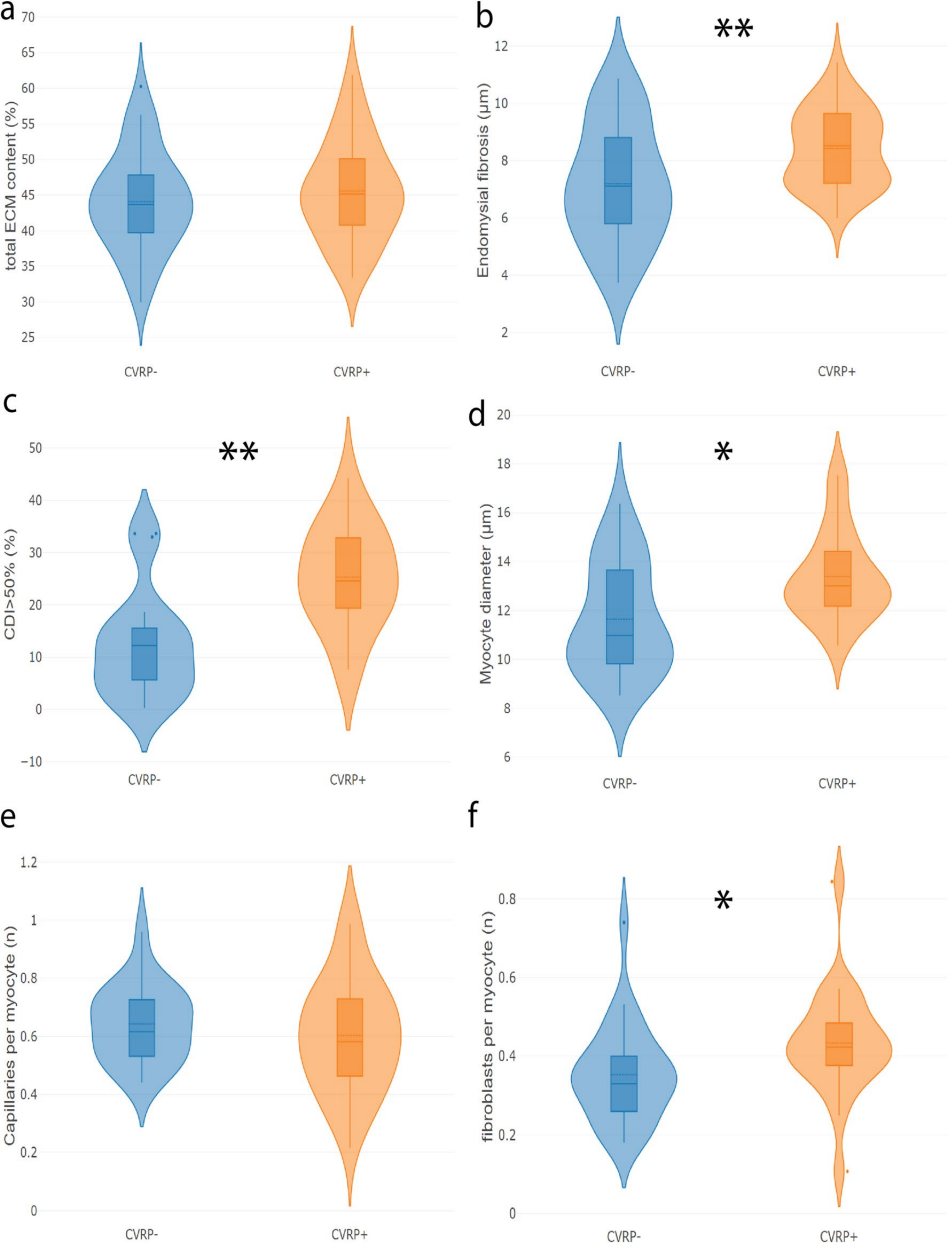
Features of structural remodeling in CVRP+ compared to CVRP– patients. 22 patients were matched and stratified into two equal groups based on their cardiovascular risk profile (CVRP). (**a**) Total ECM content is similar between the 2 groups. (**b**) Endomysial fibrosis is significantly more present in atrial tissue of CVRP+ patients (+ 1.90 µm, *p* < 0.001). (**c**) The fraction of cells that is at least 50% dissociated from its neighbors (CDI > 50%) is larger in the CVRP+ group (+ 12.6%, *p* < 0.001). (**d**) Tissue of CVRP+ patients shows signs of myocyte hypertrophy compared to CVPR- patients (+ 1.6 µm, *p* = 0.02). (**e**) The number of capillaries per myocyte was similar between both groups. (**f**) The fibroblast-to-myocyte ratio is enhanced in CVRP+ patients compared to CVRP– patients (+ 9.2%, *p* = 0.003). * significant at *p* < 0.05, ** significant at *p* < 0.001.

Structural characteristics of cardiac tissue samples of matched patients in the CVRP+ and CVRP– groups were compared. No change in total ECM content was observed (Fig. 7a), in contrast to a significant increase in inter-myocyte distance (Fig. 7b) in the CVRP+ group compared to the CVRP– group (+ 1.90 µm, *p* < 0.001). Furthermore, myocytes were bigger in CVRP+ patients compared to CVRP– patients (+ 1.60 µm, *p* = 0.02) (Fig. 7d). On average, a higher CDI was measured in CVRP+ patients (+ 9.40%, *p* < 0.001). Moreover, the fraction of cells that was at least 50% dissociated is also higher (Fig. 7c) in CVRP+ than CVRP– patients (+ 12.60%, *p* < 0.001). This expansion of endomysial fibrosis was accompanied by an increase in fibroblast density (+ 9.20%, *p* = 0.003) (Fig. 7f). No change in capillary density (− 3.70% *p* = 0.44) was observed (Fig. 7e), although absolute capillary counts were lower in CVRP+ patients (− 15% , *p* = 0.04).

### Clustering of endomysial fibrosis in patients

**Figure 8:**
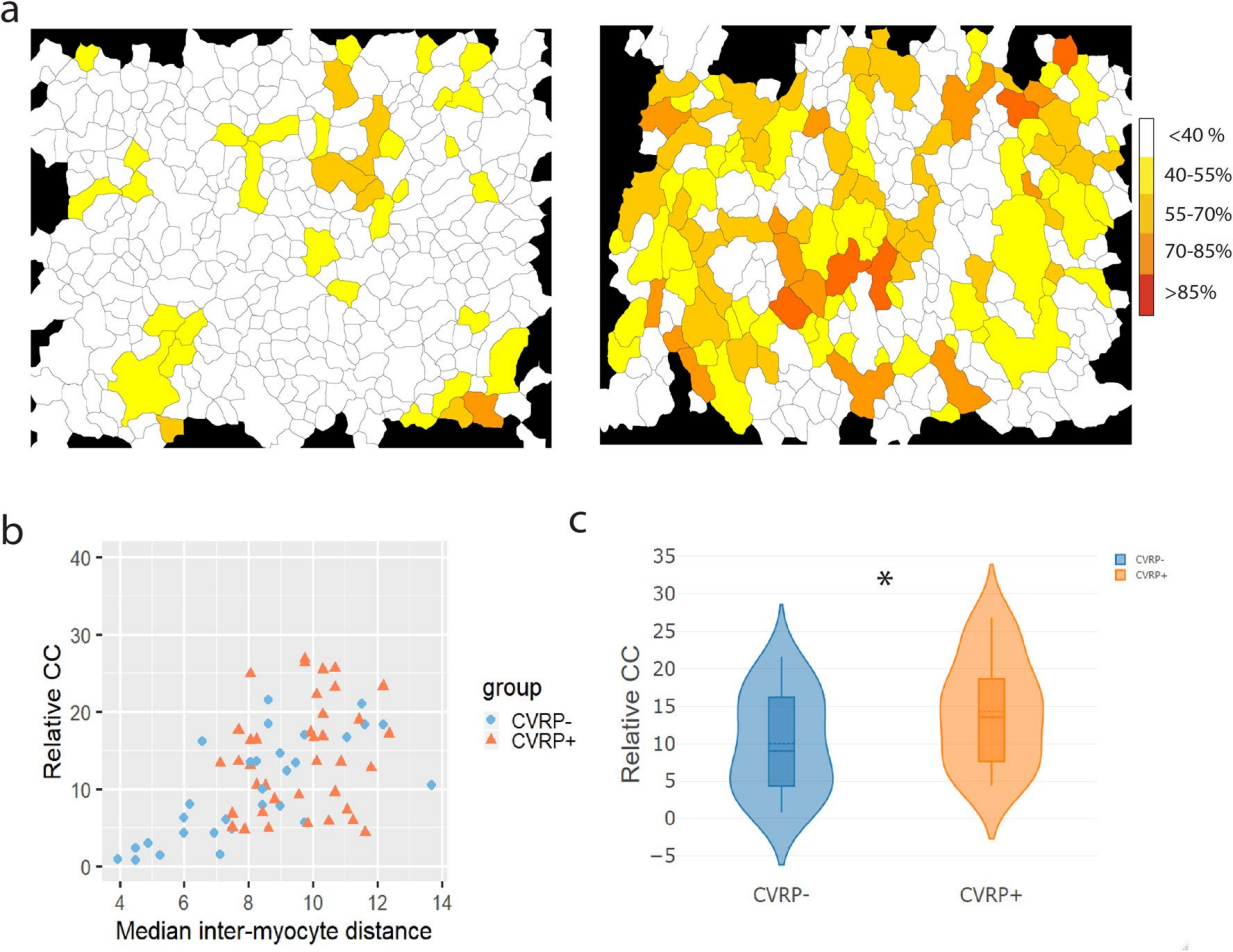
Clustering of enhanced endomysial fibrosis in CVRP– and CVRP+ patients. (**a**) Heat maps showing spatial distribution of CDI for each cardiomyocyte in an image, representing the degree of surrounding fibrosis. (**b**) Relative deviation in clustering coefficient (CC) from the expected clustering coefficient based on permutation testing. More clustering than expected in CVRP– (*p* score = 0.965) and CVRP+ (*p* score  = 0.957) patients. (**c**) The observed CC deviates more relative to the expected CC in CVRP+ patients than in CVRP– patients (+ 4.3%, *p* = 0.02). * significant at *p* < 0.05.

Visually, the spatial distribution of fibrous tissue suggested clustering of higher inter-myocyte distances, i.e. endomysial fibrosis (Fig. 8a). As an extension of the automated quantification, the degree of clustering of enhanced inter-myocyte distances was studied in both patient groups. The fraction of expected clustering coefficients that was smaller than the observed clustering coefficient was high in the CVRP– (96.5 ± 1.50%) and the CVRP+ (95.7 ± 1.44%) group. This indicates that high values of endomysial fibrosis appear in clusters, regardless of the phenotype of the patient. However, the relative increase of observed clustering coefficients compared to expected values of clustering (Fig. 8b,c) was larger in the CVRP+ group (+ 14.3 ± 1.26%) than in the CVRP– group (+ 10.0 ± 1.32%, *p* = 0.02), confirming the larger degree of spatial heterogeneity in transverse myocyte separation.

## Discussion

In the present study, simultaneous visualization and automated analysis of multiple features of structural remodeling was successfully applied. Accurate detection of cardiomyocytes and capillaries was confirmed by evaluating performance metrics. Moreover, JavaCyte offers a much faster alternative to characterize myocardial structure objectively, as it no user input is required once it started. In a validation study on goat atrial biopsies, structural properties were visualized in experimental goat models for ST-AF, LT-AF and AV-block. Inter-myocyte distance and myocyte size were increased in LT-AF, validating earlier findings in these goat models^6^. In the AV-block model, an expected increase in myocyte size was observed^19^. Outcomes from the automated quantification agreed well with manual quantification of the same sections.

In addition, applying JavaCyte, no significant inter-observer variability in providing input for automated analysis was detected in a study on human biopsies, which show more heterogeneity in tissue structure than biopsies from the goat models. Application of this novel automated analysis made it possible to compare atrial tissue from patients whose clinical profile suggested an increased risk of atrial remodeling and those whose clinical profile did not. Alterations in atrial tissue structure were detected, including more, and more clustered, endomysial fibrosis, more dissociated cardiomyocytes, proliferation of fibroblasts and cellular hypertrophy. Each of these factors has been proposed to contribute to AF progression. By contrast, similar degrees of overall fibrosis were observed in both groups.

### Advantages over traditional methods and validation in animal models

Fluorescent staining and automated analysis using JavaCyte offers several advantages over traditional approaches which apply a multi-step process involving subjective detection thresholds. Poor color contrast in conventional histology between myocytes and particularly endomysial fibrosis limits digital thresholding of images, complicating automated analysis strategies. Manual quantification of fibrosis is slow and more sensitive to larger areas of fibrosis rather than endomysial fibrosis. As a result, reports on the level of fibrosis in animal studies vary widely between and within species. In this study, fibrotic structures were visualized using WGA, a lectin that binds to sialic acid and N-acetylglucosaminyl residues in the cell membrane. WGA was previously described as a tool to stain cardiac sarcolemma and fibrotic structures^17^, as well as connective tissue and ECM. Many glycosaminoglycans in the ECM contain N-acetylglucosamin, a target of WGA^22–24^. Therefore, the use of WGA provides a suitable method to study interstitial fibrosis. This staining enabled the detection of increased endomysial fibrosis in LT-AF goats compared to ST-AF goats, particularly in the epicardium^6^, which may have been missed using Sirius Red or Trichrome staining. In prior studies using optical mapping, observations of enhanced endomysial fibrosis in LT-AF goats were linked to impaired epicardial wavefront expansion in LT-AF goats compared to ST-AF goats^7^. Applying JavaCyte, we have confirmed our earlier findings of increased endomysial thickness in LT-AF goats compared to sham-operated and ST-AF goats in the epicardial layer. Furthermore, the presence of myocyte hypertrophy was confirmed in LT-AF and AV-block goats compared to Sham-operated animals.

This approach to quantifying myocyte size and the distribution of fibrous tissue is fast, relatively inexpensive, reproducible, suitable for cryosections and allows for the simultaneous visualization of additional structures. Conventional fluorescence microscopy is limited to the detection of three different fluorescent labels at sufficiently distinct wavelengths. We used WGA in combination with staining of capillaries and fibroblasts. However, with minor modifications in the staining protocol and analysis algorithms, this method is also suitable for the quantification of other discrete structures and cell types, such as macrophages, intramyocardial smooth muscle cells and myofibroblasts with the appropriate fluorescent labels. The size and positions of all recognized structures is automatically stored, allowing further statistical analysis of the spatial intercorrelations between different structures.

Part of this potential was employed here by normalizing capillary and fibroblast counts to the number of myocytes in an image. Capillary rarefaction, i.e. a loss of the number of capillaries has been implicated in various disease processes. While a reduced capillary count per unit of surface area implies longer diffusion distances, it is an unreliable indicator for capillary rarefaction with concomitant myocyte hypertrophy. Normalization of capillary counts for the number of myocytes therefore provides important additional information on rarefaction/ angiogenesis. Furthermore, it is possible to study inter-capillary distance to investigate oxygen diffusion distances. This parameter was not analyzed in the current manuscript, but it could be calculated in a straightforward manner from the JavaCyte output files.

Moreover, fibroblasts were visualized applying an antibody directed to vimentin, the major protein in the cytoskeleton of mesenchymal cells. Vimentin-positive structures were corrected for cross-reaction with endothelial cells^21^ by subtraction. The application of a myofibroblast-specific marker such as alpha smooth muscle actin was outside the scope of this article, but could be useful to study the degree of fibroblast differentiation.

Initially, the algorithm was validated by comparing the number of automatically identified cardiomyocytes and capillaries to manual counts. The sensitivity, precision, false negative rate and F1 score were calculated as measures of precision. JavaCyte was very sensitive in recognizing cardiomyocytes and capillaries, while maintaining precision. Additionally, cardiomyocyte size and median inter-myocyte distance correlated strongly with manual measurements.

### Structural remodeling in patient biopsies

Following validation of JavaCyte in previously characterized goat models, the algorithm was applied to a clinical test case. As a result of multiple underlying comorbidities of unknown, varying durations, atrial tissue structure in patients tends to be more variable and extensive, affording a challenge to test the performance of automated quantification. One of the most important attributes of JavaCyte is standardization and reproducibility of data, independent of the operator. This quality was confirmed by studying inter-observer variation between 2 individual observers who individually took 10 photographs per sample of 10 unique patients. The images were then analyzed using JavaCyte, eliminating inter-observer variation in the data analysis phase. In this way, the impact of potential bias in area selection during microscopy and photographing of the tissue was assessed. A high degree of similarity between both analysts was observed. Moreover, JavaCyte is capable of analyzing hundreds of images per hour, making it ideally suited for high throughput analysis in large, multi-center trials in a cost-effective and reproducible manner.

In summary, cardiomyocyte size was increased in CVRP+ patients. Enhanced deposition of ECM in between cardiomyocytes was observed in patients with concomitant cardiac conditions that are believed to accelerate structural remodeling of the heart. Organized and accurate quantification of inter-myocyte distance between each pair of myocytes makes it possible to study the spatial distribution of enhanced endomysial fibrosis. In this study, the degree of dissociation of each myocyte from its neighbors was calculated (CDI), reflecting the local amount of surrounding fibrosis. A higher CDI indicated that myocytes are surrounded by more extreme endomysial fibrosis and are thus more isolated from their neighbors. The substantial rise in CDI observed in CVRP+ patients suggests that the observed increase in median inter-myocyte distance results from regions with more extreme endomysial fibrosis, rather than a mild homogenous increase of endomysial fibrosis throughout the tissue. Such a loss of inter-myocyte connections could produce zigzag conduction patterns and promote microreentry^25^. Such patterns of conduction have also previously been associated with aging^26^. Following these observations, the degree of clustering of large inter-myocyte distances was calculated. In both patient groups, large values of endomysial fibrosis clustered more often than can be expected by chance, suggesting that extensive endomysial fibrosis tends to localize in clusters. The relative degree in observed clustering compared to expected clustering of endomysial fibrosis was higher in the CVRP+ group than the CVRP– group.

Moreover, a higher fibroblast/myocyte ratio was observed in CVRP+ patients, which could imply more opportunities for electrotonic interaction between these cell types. Although a smaller number of capillaries was observed in atrial appendages of CVRP+ patients compared to CVRP– patients, the number of capillaries per myocyte and the size of these capillaries did not differ. This suggests that, although oxygen diffusion distances may have increased, capillary rarefaction does not occur under these circumstances.

## Conclusion

Here, we provide a flexible and expandable, open source tool that is well-suited for the accurate characterization of myocardial structure including the quantification of fibrous tissue distribution, myocyte hypertrophy, fibroblast density, capillary density and size, and the spatial interrelations between these features. Compared to conventional analysis, our method is highly time- and cost-efficient, and largely eliminates observer bias and inter-observer variability.

### Limitations

For experimental work described in this manuscript, frozen cardiac tissue samples were used. Although JavaCyte is only tested in frozen sections, we expect the program to work for fixed tissue slides as well, provided the staining protocol is optimized. JavaCyte was tested at magnifications ranging from 200 × to 400x. Both magnifications provide ideal cardiomyocyte size and resolution needed for accurate analysis. Depending on the microscope used, smaller magnifications may lack the required resolution to measure distances accurately, while higher magnifications will result in fewer measurements.

The analysis algorithms were developed for the quantification of structural features in regions where myocytes and capillaries are sectioned transversely. Although conduction is most rapid in the longitudinal direction of myocyte bundles, longitudinal conduction is affected less by structural remodeling. Several studies have highlighted the importance of transverse electrical uncoupling resulting from endomysial fibrosis to the discontinuous conduction and microreentry^5,6,25,27^. In addition, interstitial/ endomysial fibrosis is a major determinant for decreased myocardial compliance and diastolic dysfunction^7,8^.

Within the regions used for analysis, myocytes and capillaries were approximately, rather than exactly, transversely sectioned. For these structures, using the maximal width would represent a variable and potentially large overestimation of size. To minimize this inaccuracy, the algorithm uses the minimal Feret diameter as a measure for myocyte and capillary size. This results in a systematic but small underestimation of actual myocyte and capillary sizes, which should be similar between study groups.

Here, we have tested the method in atrial biopsies from diverse animal models (goats, pigs and horses) and human patients, but we expect that the validity and performance will be similar in transversely sectioned tissue from ventricular and skeletal muscle. Optically, the algorithms were developed to differentiate between large cells and intercellular space in transversely cut areas. Other applications of JavaCyte will be possible provided that fluorescently stained objects of interest are clearly identifiable in an image. We have not tested its performance in circumstances with very pronounced replacement fibrosis, such as ventricular infarct zones. Because infarct zones are characterized by replacement fibrosis secondary to myocyte death, we would expect that our algorithms would show more 'overall fibrosis', with fewer detectable myocytes. In this scenario, the advantage of JavaCyte analysis over conventional histology (e.g. Sirius Red or Masson's Trichrome) may be more limited, but the triple staining analyzed with JavaCyte would still yield the number of and distance between (surviving) myocytes, and the density of capillaries and fibroblasts.

CVRP+ patients were diagnosed with both persistent AF and heart failure. The pathologies responsible for heart failure in these patients were variable. It is not possible to disentangle whether the differences in tissue structure compared to CVRP– patients can be attributed to the effects associated with (end-stage) heart failure, underlying pathologies inducing heart failure, AF, or a combined synergistic effect. The impact of varying clinical profiles in distinct groups of patients should be studied in a larger, more extensively subclassified and phenotyped cohort of patients. Furthermore, the tool provided here could be used to study pathogenesis of pro-fibrotic remodeling in such a study, for example by linking histological features to mRNA and protein expression profiles.

## Supplementary information

Supplementary Information.

## References

[1] Piek A, Sillje HHW, de Boer RA (2019). The vicious cycle of arrhythmia and myocardial fibrosis. Eur. J. Heart Fail..

[2] Frangogiannis NG (2019). Cardiac fibrosis: cell biological mechanisms, molecular pathways and therapeutic opportunities. Mol. Aspects Med..

[3] Burstein B, Nattel S (2008). Atrial fibrosis: mechanisms and clinical relevance in atrial fibrillation. J. Am. Coll. Cardiol..

[4] Weber KT, Pick R, Jalil JE, Janicki JS, Carroll EP (1989). Patterns of myocardial fibrosis. J. Mol. Cell Cardiol..

[5] Kawara T, Derksen R, de Groot JR, Coronel R, Tasseron S, Linnenbank AC (2001). Activation delay after premature stimulation in chronically diseased human myocardium relates to the architecture of interstitial fibrosis. Circulation.

[6] Verheule S, Tuyls E, Gharaviri A, Hulsmans S, van Hunnik A, Kuiper M (2013). Loss of continuity in the thin epicardial layer because of endomysial fibrosis increases the complexity of atrial fibrillatory conduction. Circ. Arrhythm. Electrophysiol..

[7] Chen W, Frangogiannis NG (2010). The role of inflammatory and fibrogenic pathways in heart failure associated with aging. Heart Fail. Rev..

[8] Bench T, Burkhoff D, O'Connell JB, Costanzo MR, Abraham WT, John Sutton M (2009). Heart failure with normal ejection fraction: consideration of mechanisms other than diastolic dysfunction. Curr. Heart Fail. Rep..

[9] Krul SP, Berger WR, Smit NW, van Amersfoorth SC, Driessen AH, van Boven WJ (2015). Atrial fibrosis and conduction slowing in the left atrial appendage of patients undergoing thoracoscopic surgical pulmonary vein isolation for atrial fibrillation. Circ. Arrhythm Electrophysiol..

[10] Kohl P, Camelliti P, Burton FL, Smith GL (2005). Electrical coupling of fibroblasts and myocytes: relevance for cardiac propagation. J. Electrocardiol..

[11] Quinn TA, Camelliti P, Rog-Zielinska EA, Siedlecka U, Poggioli T, O'Toole ET (2016). Electrotonic coupling of excitable and nonexcitable cells in the heart revealed by optogenetics. Proc. Natl. Acad. Sci. USA.

[12] Wiegerinck RF, Verkerk AO, Belterman CN, van Veen TA, Baartscheer A, Opthof T (2006). Larger cell size in rabbits with heart failure increases myocardial conduction velocity and QRS duration. Circulation.

[13] Spach MS, Heidlage JF, Barr RC, Dolber PC (2004). Cell size and communication: role in structural and electrical development and remodeling of the heart. Heart Rhythm..

[14] Spach MS, Heidlage JF, Dolber PC, Barr RC (2000). Electrophysiological effects of remodeling cardiac gap junctions and cell size: experimental and model studies of normal cardiac growth. Circ. Res..

[15] Opacic D, van Bragt KA, Nasrallah HM, Schotten U, Verheule S (2016). Atrial metabolism and tissue perfusion as determinants of electrical and structural remodelling in atrial fibrillation. Cardiovasc. Res..

[16] D'Amario D, Migliaro S, Borovac JA, Restivo A, Vergallo R, Galli M (2019). Microvascular dysfunction in heart failure with preserved ejection fraction. Front Physiol..

[17] Emde B, Heinen A, Godecke A, Bottermann K (2014). Wheat germ agglutinin staining as a suitable method for detection and quantification of fibrosis in cardiac tissue after myocardial infarction. Eur. J. Histochem..

[18] Verheule S, Tuyls E, van Hunnik A, Kuiper M, Schotten U, Allessie M (2010). Fibrillatory conduction in the atrial free walls of goats in persistent and permanent atrial fibrillation. Circ. Arrhythm Electrophysiol..

[19] Neuberger HR, Schotten U, Verheule S, Eijsbouts S, Blaauw Y, van Hunnik A (2005). Development of a substrate of atrial fibrillation during chronic atrioventricular block in the goat. Circulation.

[20] Landini G, Randell DA, Fouad S, Galton A (2017). Automatic thresholding from the gradients of region boundaries. J. Microsc..

[21] Franke WW, Schmid E, Osborn M, Weber K (1978). Different intermediate-sized filaments distinguished by immunofluorescence microscopy. Proc. Natl. Acad. Sci. USA.

[22] Kostrominova TY (2011). Application of WGA lectin staining for visualization of the connective tissue in skeletal muscle, bone, and ligament/tendon studies. Microsc. Res. Tech..

[23] Ohno J, Tajima Y, Utsumi N (1986). Binding of wheat germ agglutinin in the matrix of rat tracheal cartilage. Histochem. J..

[24] Soderstrom KO (1987). Lectin binding to collagen strands in histologic tissue sections. Histochemistry.

[25] Spach MS, Boineau JP (1997). Microfibrosis produces electrical load variations due to loss of side-to-side cell connections: a major mechanism of structural heart disease arrhythmias. Pacing Clin. Electrophysiol..

[26] Spach MS, Dolber PC (1986). Relating extracellular potentials and their derivatives to anisotropic propagation at a microscopic level in human cardiac muscle. Evidence for electrical uncoupling of side-to-side fiber connections with increasing age. Circ. Res..

[27] Koura T, Hara M, Takeuchi S, Ota K, Okada Y, Miyoshi S (2002). Anisotropic conduction properties in canine atria analyzed by high-resolution optical mapping: preferential direction of conduction block changes from longitudinal to transverse with increasing age. Circulation.

