# Supplementary material for: JavaCyte, a novel open-source tool for automated quantification of key hallmarks of cardiac structural remodeling
Source: Sci Rep. 2020 Nov 18;10:20074. doi: 10.1038/s41598-020-76932-3 (PMC7675975; doi:10.1038/s41598-020-76932-3)
Supplement: Supplementary file 1 — Supplementary Information. [file 41598_2020_76932_MOESM1_ESM.docx]

Supplement 1: JavaCyte, a novel open-source tool for automated quantification of key hallmarks of cardiac structural remodeling

Authors: Winters J.^1^, Edler von Braunmuhl M., Zeemering S.^1^, Gilbers M.^1^, Ten Brink T.^1^, Scaf B.^1^, Guash E.^2-4^, Mont L.^2-4^, Batlle M.^2^, Sinner M^10^., Hatem S.^5^, Mansour MK^6^ , Fabritz L.^7,8^, Sommerfeld L.^7^, Kirchhof P.^7-10^, Isaacs A^1^., Stoll M^11^., Schotten U.^1^, Verheule S*.^1^

**Protocol for WGA-cd31/GSIB4-Vimentin**Preparation: tissue samples were obtained and snap frozen in liquid nitrogen. Tissue was cut into 6 µm slices with a cryotome (22°C )and stored at -80°C.

1. Air-dry slides for 30 min at room temperature (RT):
2. Fixate with -20°C fresh acetone for 10 min
3. Remove slides from acetone and air dry at RT until all acetone has evaporated.
4. Wash 2x in 0.1M PBS (1xPBS)
5. Block with blocking solution (Blocking solution: dissolve 2m/v% fraction V BSA and 0.3M glycine in 1xPBS) for 60 minutes.
6. Primary antibodies
   1. In case of WGA-CD31-Vimentin staining: Incubate slides with the primary antibody for Vimentin and CD31, overnight at room temperature.
      1. Mouse anti human CD31 (Dako): 1/50
      2. Mouse monoclonal anti-vimentin ab92547 (Abcam): 1/150.
   2. In case of WGA-GSIB4-Vimentin staining:
      1. Mouse monoclonal anti-vimentin ab92547: 1/150
7. Next morning: wash 2 x 10 minutes with 1xPBS
8. Incubate with secondary antibody solution for 120 minutes at RT:
   1. In case of WGA-CD31-Vimentin
      1. Goat anti-rabbit IgG (H+L) cross-adsorbed secondary antibody, Alexa Fluor 405 (ThermoFisher): 1/200
      2. Wheat germ agglutinin, Alexa Fluor 594 conjugate (ThermoFisher): 1/200
      3. Goat anti-mouse IgG (H+L) highly cross-adsorbed secondary antibody, Alexa Fluor 488 (ThermoFisher): 1/200
   2. In case of WGA-GSIB4-Vimentin:
      1. Goat anti-rabbit IgG (H+L) cross-adsorbed secondary antibody, Alexa Fluor 405 (ThermoFisher): 1/200
      2. Wheat germ agglutinin, Alexa Fluor 594 conjugate (ThermoFisher): 1/200
      3. Isolectin GS-IB4 Griffonia simplicifolia, Alexa Fluor 488 conjugate (ThermoFisher)
9. Wash 3 times 10 minutes in 1xPBS
10. Dry for 5 minutes
11. Cover slip with Prolong Gold antifade mounting medium
12. Store at -4°C for short periods of time (up to 2 weeks) or -20° for longer periods of time.
